# Diagnostic Challenge of Small Bowel Neuroendocrine Tumor in a Young Female Patient

**DOI:** 10.7759/cureus.37925

**Published:** 2023-04-21

**Authors:** Somin Lee, Abhilasha Jyala, Haider Ghazanfar, Dongmin Shin, Harish Patel

**Affiliations:** 1 Internal Medicine, BronxCare Health System, Bronx, USA; 2 Gastroenterology, BronxCare Health System, Bronx, USA

**Keywords:** well-differentiated neuroendocrine tumor, rare cause of acute abdominal pain, young female with abdominal pain, ileum, ileal neuroendocrine tumor

## Abstract

Neuroendocrine tumors (NETs) are rare cancers arising from neuroendocrine cells and are characterized by their ability to secrete functional hormones causing distinctive hormonal syndromes. The incidence of NET has increased over the years, and small bowel neuroendocrine tumor (SBNET) is one of the most challenging to detect due to its varied presentation and poor accessibility with traditional endoscopic methods. Patients with SBNET present with variable hormonal symptoms, such as diarrhea, flushing, and nonspecific abdominal pain, which often delay the diagnosis. We present the case of a young patient who underwent multidisciplinary workups leading to a successful diagnosis of SBNET promptly. The patient was a 31-year-old female who presented to the emergency department with complaints of nausea, vomiting, and sudden-onset, severe, sharp abdominal pain. CT scan of her abdomen showed an area of irregular intraluminal soft tissue density suspicious for a mass in the mid-small bowel. The patient’s initial enteroscopy was normal. A video capsule endoscopy showed a small bowel mass, which was consistent with SBNET confirmed by pathology later. This case emphasizes the importance of considering SBNET as a differential diagnosis in young patients with nonspecific symptoms of abdominal pain and highlights the role of multidisciplinary approaches in achieving prompt diagnosis and treatment.

## Introduction

Neuroendocrine tumors (NETs) arise from neuroendocrine cells and are characterized by their ability to secrete functional hormones throughout the body causing distinctive hormonal syndromes or nonspecific abdominal pain which delays the diagnosis. Small bowel neuroendocrine tumor (SBNET) is challenging to detect due to its extremely low incidence, various clinical presentation, and poor accessibility of the distal small bowel with the traditional endoscopic method [1]. Here, we share our diagnostic challenge in a case of a young patient who underwent multidisciplinary workups leading to a successful diagnosis of SBNET promptly.

## Case presentation

A 31-year-old female presented to the emergency department with complaints of nausea, vomiting, and abdominal pain for one day. The abdominal pain was sudden in onset, sharp in character, severe in intensity, localized to the periumbilical region, radiating to the back, not related to eating, and without any specific relieving factors. It was associated with one episode of nonbilious nonbloody vomiting. She denied diarrhea, constipation, weight loss, hematochezia, and melena. Her past medical history was significant for left internal jugular vein thrombus, left transverse and sigmoid sinus thrombosis, and subdural hematoma. She had a history of abdominoplasty 12 years ago. She denied any family history of gastrointestinal malignancies. She denied smoking cigarettes or using any recreational drugs. She drank alcohol socially. She had been taking apixaban for left internal jugular vein thrombosis.

At the time of presentation, she was found to have a blood pressure of 133/75 mmHg, a heart rate of 85 beats per minute, a temperature of 36.7°C, a respiratory rate of 14 breaths per minute, and oxygen saturation of 99% at room air. She had bilateral vesicular breathing and normal heart sounds. She had scars on the abdomen from her previous surgery, mild abdominal tenderness in the periumbilical region, and normoactive bowel sounds. The patient’s initial laboratory findings are summarized in Table 1.

**Table 1:** The patient’s initial laboratory findings.

Laboratory parameter	Value	Reference range
White blood cell count	7.5	4.8–10.8 k/µl
Hemoglobin	11.9	12.0–16.0 g/dL
Hematocrit	36.4	42.0–51.0%
Mean corpuscular volume	83.4	80.0–96.0 fL
Platelet	242	150–400 k/µL
Sodium	137	135–145 mEq/L
Potassium	4.5	3.5–5.0 mEq/L
Bicarbonate	27	24–30 mEq/L
Chloride	100	98–108 mEq/L
Glucose	89	70–120 mg/dL
Blood urea nitrogen	8.0	8.0–26.0 mg/dL
Creatinine	0.8	0.5–1.5 mg/dL
Calcium	9.1	8.5–10.5 mg/dL
Albumin	4.6	3.4–4.8 g/dL
Total bilirubin	0.3	0.2–1.2 mg/dL
Direct bilirubin	<0.2	0.0–0.3 mg/dL
Alkaline phosphatase	75	53–128 U/L
Aspartate transaminase	21	9–48 U/L
Alanine aminotransferase	32	5–40 U/L
Total protein	6.8	6.0–8.5 g/dL

**Figure 1:**
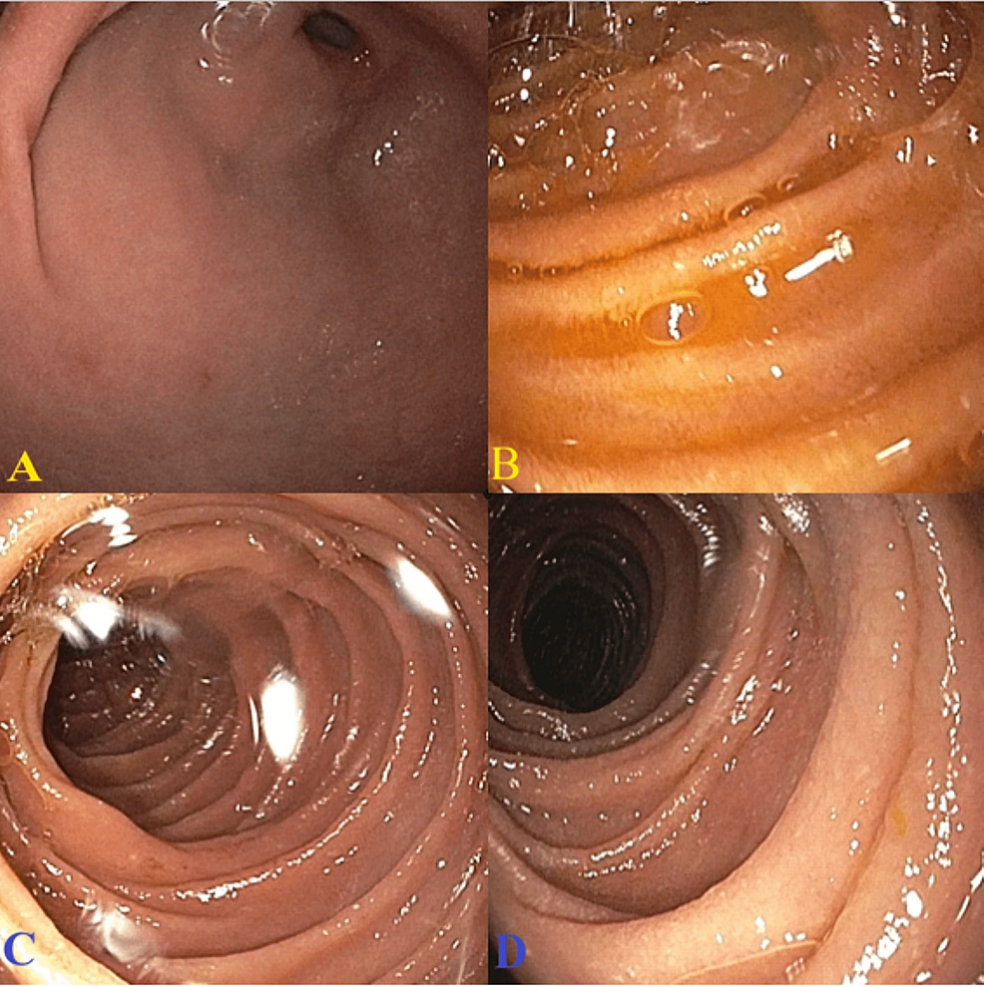
Enteroscopy showing (A) the antrum of the stomach, (B) the second part of the duodenum, (C) the fourth part of the duodenum, and (D) the jejunum.

She underwent CT of the abdomen and pelvis with contrast which showed an area of irregular intraluminal soft tissue density suspicious for a mass in the mid-small bowel with adjacent multiple enlarged lymph nodes and a smaller 7 mm stellate-like lesion within the mesentery. The patient’s carcinoembryonic antigen (CEA) was 0.6 ng/ml (reference range = ≤5.0 ng/mL), cancer antigen 19-9 (CA 19-9) was 2.7 (reference range = 0.0-37.0 U/mL), and cancer antigen 125 (CA-125) was 7.5 (reference range = ≤35.0 U/mL). She underwent an enteroscopy which showed no gross lesion in the esophagus, stomach, duodenum, and proximal and mid-jejunum (Figure 1).

**Figure 2:**
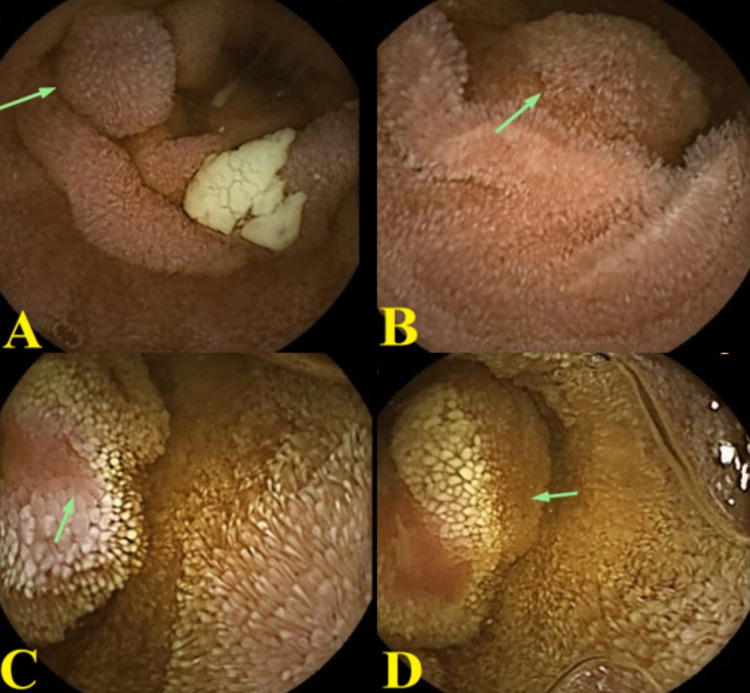
Video capsule enteroscopy showing (A) a polyp in the distal duodenum, (B) a polyp at the mid-jejunum, (C) a mass lesion in the distal ileum, and (D) a mass lesion in the distal ileum.

In light of abnormal CT findings but normal enteroscopy, she was planned for a video capsule endoscopy. Video capsule enteroscopy showed several polyps throughout the small bowel. It also showed a medium-sized mass without bleeding found in the distal ileum of the small bowel after three hours and 17 minutes of capsule ingestion (Figure 2).

She underwent diagnostic laparoscopy. About 160 cm proximal to the terminal ileum, a puckered firm small bowel mass was seen with thickened adjacent mesentery. A mini-laparotomy was done which showed a 2 cm small bowel mass in the small intestine and a 3 cm mass in the associated mesentery. The mass was resected with 10 cm proximal and distal margins to include the associated mesentery. A side-to-side isoperistaltic primary small bowel anastomosis was done. A small bowel mass with the associated mesentery was sent to pathology. Pathology showed invasive, well-differentiated, Grade 1 neuroendocrine neoplasm which was invading through the muscularis propria into the submucosal tissue without penetration of the overlying serosa. All surgical margins were found to be free of the neoplasm. The metastatic neoplasm was found to be present in four out of eight mesenteric lymph nodes. The neoplasm was found to be positive for chromogranin, synaptic ficin, CD56, CDX2, and Ki-67 index of approximately 1%. It was negative for CK7, DK20, PAX8, and TTF-1. Her serum chromogranin A was 213 (normal <311), serum serotonin was 257 ng/mL (reference range = 56-244 ng/mL), and 24-hour urine 5-hydroxyindolacetic acid level (5-HIAA) was 2.6 mg/24 hours (reference range = ≤6.0 mg/24 hours). The patient was discharged with outpatient follow-up with oncology, surgery, and gastroenterology.

## Discussion

In 1907, Oberndofer first described these tumors as carcinoids [1]. The primary site of NET can be the lung, appendix, cecum, colon, liver, pancreas, rectum, small intestine, or stomach. According to the national Surveillance, Epidemiology, and End Result (SEER) program registry, the incidence of NET has increased 6.4-fold from 1973 to 2012 from 1.09 per 100,000 to 6.98 per 100,000. The annual incidence of SBNET in the United States was 1.05 per 100,000 persons in 2012 [2]. NETs account for less than 2% of gastrointestinal (GI) cancers [3]. It is uncertain but assumed that the increasing incidence of NET is probably related to the growing use of imaging, endoscopy, and improved understanding by physicians. SBNET refers to the anatomically arising NET in the small bowel from the ligament of Treitz to the ileocecal valve [4]. NET usually occurs in the sixth decade of life, with the median age at diagnosis being 63 years. The overall NET prevalence in women is 52% and in men is 48% from a total of 35,618 patients with NET in the SEER database. Males are more likely to have it in the jejunum and ileum. In addition, jejunal and ileal SBNETs are more frequently reported in white patients (17%) and African American patients (15%), which is significantly higher than the occurrence in Asian/Pacific Islander and American Indian/Alaskan Native patients (p < 0.001) [1]. In contrast, rectal NETs are more prevalent in Asian/Pacific Islander patients (41%), American Indian/Alaskan Native patients (32%), and African American patients (26%) compared to white patients (12%) (p < 0.001) [1].

The carcinoid syndrome, described as flushing, diarrhea, valvular heart disease, and bronchospasm, is caused by the excess secretion of neuroendocrine hormones. However, without hepatic metastasis of NET, most of the confined locoregional SBNET do not present this typical carcinoid syndrome due to the excess hormones being metabolized and inactivated by the liver [4,5]. Most patients are asymptomatic for a long period of time or present with nonspecific symptoms of abdominal pain treated as irritable bowel, allergy, stress, or food-related [6]. These variable and nonspecific symptoms of SBNET delay the diagnosis. The median time of symptom onset to diagnosis can vary from 4.3 months to 9.2 years [3,7]. SBNETs are often advanced with local nodal metastasis at the time of diagnosis. The local invasion often causes fibrosis of the mesentery, resulting in bowel obstruction, ischemia, perforation, or bleeding, requiring emergent surgery [4].

Diagnostic modality

The diagnostic process of SBNET should be individualized by the patient’s presentation. For example, patients presenting with carcinoid symptoms of flushing and diarrhea may benefit by promptly testing the biochemical markers for SBNET [5].

Neuroendocrine cells produce hormones or amines that can be used as screening serum biochemical markers, such as chromogranin A (CgA), CgB, bradykinin, substance P, neurotensin, human chorionic gonadotropin, neuropeptide K, and neuropeptide PP [6]. Among these, CgA is the best overall screening biomarker because it is secreted by a wide variety of NETs, regardless of primary sites, including nonfunctional tumors. Moreover, CgA is a sensitive and specific marker for NETs and correlates with both tumor volume and prognosis [4,6]. According to a recent meta-analysis of 13 studies, CgA has a sensitivity of 73% and a specificity of 95% for the diagnosis of NET [8]. Serotonin-secreting NETs can be diagnosed using the biomarker 5-HIAA, a serotonin breakdown product. Compared to the serum serotonin level, which changes throughout the day based on activity and stress levels, urinary 5-HIAA is more useful and reliable as it has an 88% specificity [3].

Imaging such as CT may be used as the first step in the investigation of patients with obstructive symptoms or it can be discovered after an emergent surgery [5]. Anatomical and functional images can be used for the diagnosis of SBNET. Anatomical imaging includes CT and MRI whereas functional imaging includes octreotide scan and positron emission tomography (PET). Anatomic imaging is useful for localizing tumors and measuring tumor burden and evaluating the option of surgical resection, whereas functional imaging has a higher sensitivity and helps find occult metastases and monitor recurrence surveillance [5]. CT is the most common and widely available imaging modality. It provides a wide anatomical view of the chest, abdomen, and pelvis, including vascular and lymph nodes, but the extent of liver metastasis is frequently underestimated compared to MRI [3,5]. CT has 83% of high sensitivity and 76% of specificity in diagnosing NET [3]. MRI has several benefits compared to CT. MRI has 93% sensitivity and 86% specificity for detecting NET. Additionally, MRI avoids ionizing radiation exposure and improves the detection of liver metastases [3]. According to a study comparing CT and MRI, the sensitivity for detecting liver metastases was found to be 78.5% and 95.2%, respectively [5]. In patients who may need liver resection, this can make a significant difference. In MRI, tumors can be more visible and assessable using a specific contrast agent (gadoxetic acid or gadopentetic acid-based gadolinium) [3].

Functional imaging techniques utilize the fact that most NETs express somatostatin receptors (SSTRs). The somatostatin analogs, mostly pentetreotide or lanreotide, are attached to indium, an isotope, that emits single-photon emission tomography (^111^indium-octreotide, octreoscan). This indium-attached somatostatin analog, radiolabeled SSA, binds to SSTR2, 3, and 5 expressed on NET and localizes NET [3,5]. It can provide functional information about the tumor. The overall sensitivity of ^111^indium-octreotide is 52-78% and the specificity is 98% for the detection of NET [3,6]. Octreotide scanning can also be used as a follow-up modality to assess response to octreotide treatment. However, it is limited if the tumor does not express SSTR and does not have an affinity to the tracer [3]. In the last decades, gallium-68-based imaging has been favored over ^111^In-SRS for the choice of functional imaging for SBNETs as ^111^indium-octreotide sensitivity is lower for primary SBNET [5]. Many Ga-labeled ligands are available, namely, ^68^Ga-DOTATATE, ^68^Ga-DOTATOC, and ^68^Ga-DOTANOC, with each having different affinities to different SSTR subtypes. By several criteria, ^68^Ga-PET is superior to ^111^In-SRS imaging, including reduced radiation exposure, faster acquisition time, higher spatial resolution, and accuracy. According to meta-analyses, ^68^Ga-PET has a mean sensitivity of 88-93% for the detection of NET, and PET ^68^Ga-DOTATOC and ^68^Ga-DOTANOC report 92-93% specificity [3-5].

Endoscopic ultrasonography (EUS) and standard axial endoscopy are essential for the diagnosis and treatment of gastro-entero-pancreatic NETs. Upper and lower GI endoscopies (standard axial endoscopy) are crucial for the detection, biopsy, and therapeutic resection of GI NETs (stomach, duodenum, rectal, colon). EUS is the diagnostic gold standard for pancreatic NET [9]. However, due to poor accessibility in the distal small intestine, standard axial endoscopy and EUS often fail to detect the small intestine lesions and delay the diagnosis of SBNET. Capsule endoscopy and double balloon enteroscopy enable direct visualization of the entire small bowel, improving the diagnostic yield for SBNET [10]. There is scant data on the efficacy and safety of these approaches. Due to its rarity, the data are usually based on small retrospective studies, and limited data on sensitivity and specificity are currently available. Instead, diagnostic yield is often used in studies [9,10]. Capsule endoscopy is a patient-friendly and minimally invasive method for visualizing the entire small bowel. It has reported a diagnostic yield of 45-72% in SBNET [10]. A study compared capsule endoscopy and other diagnostic images, CT, and MRI. Capsule endoscopy revealed a higher diagnostic yield in detecting small lesions (p < 0.001), concluding capsule endoscopy to be a superior diagnostic technique to CT and MRI [11]. The main limitation of capsule endoscopy is its inability to collect biopsy samples or perform a therapeutic procedure. CE can also miss submucosal lesions in the small bowel. Capsule retention is considered the major complication in 1.5-2.6% of cases [10]. Double balloon enteroscopy shows a variable diagnostic yield ranging from 30% to 80%, but a false-positive rate of 17% has been reported [9]. Due to the lower rate of false positives in capsule endoscopy, it should be the first choice for SBNET endoscopy diagnosis. Double balloon enteroscopy should be performed in patients with abnormal capsule endoscopy to take a biopsy for definitive diagnosis and tattooing before surgery, or in case of contraindication for capsule endoscopy, for example, known intestinal stenosis [9,10].

Although biochemical markers, imaging, and endoscopy suggest NET, a pathologic confirmation of surgical specimens or biopsies is required [4]. The histologic suggestion of a well-differentiated NET with cells arranged in nest patterns, salt, and pepper chromatin, and amphophilic cytoplasm is a characteristic feature of NET histology. CDX2, PAX6, ISL1, and TTF-1 positivity can suggest a primary origin of NET. CDX2 positivity indicates the small bowel as the primary site [4,12].

Treatment and prognosis

Surgical resection is the preferred first-line treatment of SBNET. The goals of surgical resection are curative resection of the primary, regional lesions, and distant metastatic disease with cytoreductive intent along with palliative resection for symptom relief by removing tumor-releasing bioactive agents [6,13]. Surgical management improves survival by improving disease clearance and reducing the risk of developing metastasis. Median overall survival of 9.5 years versus 5.3 years in the elective prophylactic surgery group versus the delayed or nonsurgical group (six months after diagnosis) has been reported [14]. Although this study had heterogeneity bias, the delayed nonsurgical group was older and likely to have metastatic hepatic and extrahepatic lesions [13,14]. However, 58% (53/91) of the delayed nonsurgical group eventually received surgery at some point, either primary resection or emergently due to developing obstructive symptoms. The necessity of locoregional resection and cytoreduction of tumors by surgery cannot be overlooked [13,14]. The SEER database reports better survival is linked with at least one lymph node removal than no lymph node removal (hazard ratio = 0.64, p = 0.0027) [13]. The current North American Neuroendocrine Tumor Society guidelines recommend routine lymph node clearance with primary tumor resection. The gold standard surgical management SBNETs is open laparotomy. The surgeon should manually palpate and inspect the entire length of the small bowel to identify small and often subcentimeter and multifocal SBNET resecting the primary tumors, regional lymph nodes, mesenteric masses, and peritoneal metastases [13].

Several systemic treatment options are available for the treatment of NET, including SSAs, everolimus, and peptide receptor radionuclide therapy (PRRT) [4]. SSAs have been used for a long time as a systemic treatment of NET. SSAs are the first-line treatment for functional and nonfunctional metastatic SBNETs due to their antiproliferative effect and control of carcinoid symptoms. SSAs alleviate carcinoid syndrome and have an antiproliferative activity that improves progression-free survival (PFS) than placebo (14.3 months vs. 6 months) [4,5]. Everolimus is a mammalian target of rapamycin inhibitor which is only approved in progressive nonfunctional NET. It has shown improved median PFS when used as monotherapy compared with placebo (11.0 months vs. 3.9 months) [4]. PRRT delivers therapeutics by using radiolabeled SSA to SSTR-expressed cells, selectively targeting NET cells [6]. PPRT is the preferred second-line treatment for patients who experience disease progression while receiving SSA [4].

Due to its extreme rarity with an annual incidence of 1.05 per 100,000 persons [2], variable presentation, and poor accessibility of the distal small bowel with the standard endoscopic procedure, SBNET is difficult to diagnose [4,5]. These delays result in advanced-stage SBNET at the time of diagnosis. A multicentric lesion and metastatic spread to the regional lymph node are often found with SBNET. In population-based studies, small intestinal NETs are metastatic upon presentation in about 30% of patients [15]. Small intestine NETs tend to have high morbidity and mortality because of metastatic burden, mesenteric fibrosis leading to ischemia, and surgical emergency [12]. Compared to other GI carcinoids, SBNET carcinoids have a low five-year survival rate (60.5%). With hepatic metastasis, the five-year survival rate decreases to 18-32% [6,15]. The overall five-year survival rate is reported to be 67.2% for patients with gastrointestinal carcinoids. Each GI carcinoid’s overall five-year survival rate is stomach 81%, appendix 98%, colon 62%, and rectum 87% [6]. This diagnostic challenge of SBNET increases the risk of patient presentation of a surgical emergency and can relate to the overall poor prognosis compared to other GI NETs [8]. Malignant features of SBNETs are assessed by multiple factors, including the size of the tumor, local spread, depth of invasion, the extent of metastases at the time of diagnosis, mitotic rate, multiplicity, and the presence of carcinoid syndrome. Ki-67, proliferation markers, and mitotic rate can be used as criteria for the classification and grading of NET [4,12].

Although complete resection of tumors seems to be curative, long-term recurrence rates of about 50% have been reported [5]. Close surveillance for 10 years after the resection of NET should be followed at intervals of six to 12 months with imaging, biochemical markers, or endoscopy. Currently, surveillance strategies are varied in clinical practice, and it is recommended that they are tailored to individual presentations [1,5,6].

## Conclusions

NETs arise from neuroendocrine differentiation and are characterized by their ability to secrete functional hormones throughout the body causing distinctive hormonal syndromes. SBNET is challenging to detect due to its extremely low incidence, varied presentation, and poor accessibility of the distal small bowel with traditional endoscopic methods. Patients suspected of having SBNETs should have an individualized diagnostic process involving testing biochemical markers or obtaining functional or anatomical imaging based on clinical presentation. Upper and lower GI endoscopies (standard axial endoscopy) are crucial for the detection, biopsy, and therapeutic resection of GI NETs (stomach, duodenum, rectal, colon). Surgical resection is the preferred first-line treatment of SBNET. The goal of surgical resection should be sufficient curative resection of the primary, regional lesions, and distant metastatic disease with cytoreductive intent along with palliative resection for symptom relief by removing bioactive agents releasing tumor. Several systemic treatment options are available for the treatment of NET, including SSAs, everolimus, and PRRT. Although complete resection of tumors seems to be curative, it has been noted that long-term recurrence rates are about 50%. Close surveillance for 10 years after the resection of NET should be followed at intervals of six to 12 months with imaging, biochemical markers, or endoscopy.
